# Leadership at the Nexus: Culture, Spirituality, and Religiosity as Strategic Determinants of Positive Clinical Work Environments

**DOI:** 10.1155/jonm/1927647

**Published:** 2026-05-09

**Authors:** Jacqueline Maria Dias, Jonas Cruz, Aurelija Blaževičienė, Martin Červený

**Affiliations:** ^1^ Department of Nursing, College of Health Sciences University of Sharjah, Sharjah, UAE; ^2^ Department of Medicine, School of Medicine, Nazarbayev University, Astana, Kazakhstan, nu.edu.kz; ^3^ Head of Department of Nursing, Faculty of Nursing, Medical Academy, Lithuanian University of Health Sciences, Eivenių g. 2, Kaunas, LT-50161, Lithuania, lsmuni.lt; ^4^ Pavol Jozef Šafárik University in Košice, Košice, 040 11, Slovak Republic, upjs.sk

**Keywords:** clinical, mentorship, religiosity, spirituality, workplace culture, work environments

## Abstract

**Aim:**

To synthesize the contributions of this special issue examining how workplace culture, spirituality, religiosity, and mentorship shape positive clinical work environments and inform contemporary nursing management.

**Background:**

Amid workforce shortages, rising clinical complexity, and escalating moral demands, nursing management is often evaluated through operational metrics that fail to capture the ethical and relational foundations of sustainable practice. Growing evidence highlights the importance of organizational culture, meaning‐making, and inclusive leadership in supporting nurse retention and well‐being.

**Design:**

Editorial synthesis of contributions within the special issue.

**Results:**

The papers demonstrate that workplace culture is enacted through everyday leadership practices, ethical clarity, and relational consistency, directly influencing psychological safety and professional commitment. Spirituality is framed as a professional resource supporting meaning‐making and resilience when reflection and dialog are enabled. Religiosity and cultural diversity require culturally responsive leadership to foster trust and inclusion. Mentorship emerges as a strategic leadership function that strengthens confidence, moral coherence, and retention. Collectively, the contributions shift attention from individual resilience to organizational stewardship of ethical and relational infrastructure.

**Conclusions:**

Positive work environments are intentionally shaped through leadership that governs culture, inclusion, and mentorship alongside operational performance.

**Implications for Nursing Management:**

Nurse managers must extend their focus beyond operational performance to stewardship of culture and ethical climate. Workplace culture, spirituality, religiosity, and mentorship are structural drivers of psychological safety, retention, and professional commitment. Leaders should foster inclusive, respectful environments that support ethical dialog and cultural humility and formalize mentorship as a strategic workforce priority. Embedding values‐based and relational leadership within governance frameworks is essential for sustaining workforce resilience.

## 1. Background

Healthcare systems are currently characterized by structural workforce shortages, intensifying clinical complexity, and escalating moral and emotional demands on nurses [1, 2]. In this environment, nursing management is frequently evaluated through operational metrics—efficiency, throughput, and cost containment [3]. Yet such indicators inadequately capture the conditions under which professional sustainability is either enabled or undermined [4]. The contemporary challenge for nursing management is therefore not only operational coordination but also governance of the ethical and relational infrastructure that sustains nursing work [5].

This special issue—*The Nexus of Workplace Culture, Spirituality, and Religiosity in Fostering Positive Clinical Work Environments for Nurses*—positions workplace culture, spirituality, religiosity, and mentorship as interdependent determinants of positive clinical environments. Taken collectively, the contributions move beyond individual‐level explanations of resilience and instead foreground the organizational conditions under which nurses are able to sustain meaning, moral coherence, and professional commitment.

Workplace culture is widely acknowledged as central to nurse retention, engagement, and quality of care. Yet culture is often discussed at a distance from practice. It becomes visible in moments of pressure: how ethical concerns are handled, whether emotional labor is recognized, and how difference is accommodated rather than managed away. Spirituality and religiosity is understood in inclusive and nonprescriptive ways are part of this landscape. For many nurses, they inform how suffering is interpreted, how resilience is sustained, and how meaning is found in demanding clinical roles. Mentorship, meanwhile, represents one of the most practical ways through which these values are negotiated and carried forward within organizations. The contributions in this special issue make clear that positive work environments are neither incidental nor culturally neutral. They are shaped through everyday leadership practices and organizational decisions.

Several papers focus on workplace culture as it is experienced by nurses in real clinical settings. These studies show that cultures characterized by respect, ethical clarity, and relational leadership are associated with stronger professional commitment and a greater sense of moral coherence. Importantly, they also illustrate that culture is enacted, not declared. As many nursing leaders will recognize from their own experience, it is shaped in routine interactions; including how concerns are responded to, how fairness is demonstrated, and how consistently values are upheld when resources are stretched. Where leadership is visible and trustworthy, nurses describe greater psychological safety; where it is inconsistent, distress, and disengagement are more likely to take root.

Other contributions explore spirituality as a professional resource rather than a personal attribute. Across diverse contexts, spirituality is described in terms of meaning‐making, connectedness, and purpose in nursing work. The papers suggest that nurses are better able to navigate moral distress and emotional strain when organizational cultures allow space for reflection and ethical dialog. This does not imply the promotion of specific beliefs. Rather, it reflects leadership practices that acknowledge the existential dimensions of care—something many nurse managers recognize intuitively but struggle to formalize within busy clinical environments. Structured reflection, supportive supervision, and attentive leadership presence emerge as practical responses.

Religiosity and cultural diversity are also addressed, reflecting the realities of increasingly pluralistic healthcare workforces. Several papers examine how religious identity intersects with workplace culture through accommodation practices, team relationships, and ethical decision‐making. The findings highlight the central role of nurse managers in balancing organizational consistency with respect for difference. Leaders who approach these situations with cultural humility and fairness are more likely to foster trust and cohesion. Conversely, ambiguity, or avoidance can contribute to tension and perceptions of exclusion, an observation familiar to many in leadership roles.

Mentorship appears throughout this special issue as a critical connecting thread. Beyond its developmental function, mentorship operates as a cultural and ethical anchor within organizations. Effective mentorship provides nurses—particularly those at early or transitional stages of their careers with space to make sense of professional expectations, ethical challenges, and organizational values. Several papers demonstrate how structured mentorship supports confidence, psychological safety, and retention. Equally telling are accounts of environments where mentorship is absent or informal, leaving nurses to navigate moral and professional challenges in isolation. For nursing management, these findings reinforce the need to treat mentorship as a strategic leadership responsibility rather than a discretionary activity.

Collectively, the papers in this special issue point to important implications for nursing management. Leadership in contemporary healthcare requires engagement with the ethical, cultural, and meaning‐centered dimensions of work, alongside operational and performance demands. This includes developing capability in values‐based leadership, cultural responsiveness, and mentorship, all of which are increasingly essential to workforce sustainability. Many nurse leaders will recognize these competencies as central to their role, even if they are not always explicitly named or supported within organizational frameworks.

At the organizational and policy level, the evidence presented here challenges approaches to staff well‐being that focus narrowly on individual resilience. Instead, it supports a more integrated view, one that recognizes ethical climate, cultural inclusion, spirituality, and mentorship as interdependent features of healthy work environments. Embedding these considerations within leadership development programs, workforce policies, and quality frameworks is necessary if organizations are to respond meaningfully to ongoing workforce challenges.

## 2. Conclusion and Future Directions

At this nexus lies a critical reorientation of nursing management. The task is not only to manage performance but also to steward the moral and relational foundations of practice. Culture, spirituality, religiosity, and mentorship together constitute the institutional infrastructure through which nurses sustain purpose, belonging, and commitment [6]. Recognizing and governing this infrastructure is not an aspirational endeavor; it is central to the legitimacy and future resilience of nursing leadership.

## Author Contributions

Jacqueline Maria Dias conceived, initiated, and wrote the first draft of this editorial. Jonas Cruz, Aurelija Blaževičienė, and Martin Červený revised and reviewed the manuscript.

## Funding

No funding was received.

## Disclosure

All authors approved the final draft of the manuscript.

## Ethics Statement

The authors have nothing to report.

## Consent

The authors have nothing to report.

## Conflicts of Interest

The authors declare no conflicts of interest.

## Data Availability

No datasets were generated or analyzed during the current study.
